# Supplementary material for: Genetic Structuring of One of the Main Vectors of Sylvatic Yellow Fever: Haemagogus (Conopostegus) leucocelaenus (Diptera: Culicidae)
Source: Genes (Basel). 2023 Aug 24;14(9):1671. doi: 10.3390/genes14091671 (PMC10531017; doi:10.3390/genes14091671)
Supplement: Supplementary file 1 [file genes-14-01671-s001.zip › genes-2537643-supplementary.pdf]

Supplementary Materials

Table S1. Locations and numbers of adult specimens and immature forms of *Hg. leucocelaenus* collected to analyze single nucleotide polymorphisms (SNPs).

Date	Location	Description	Gender	Form
			F	Adult
			F	Adult
	Balsamo		F	Adult
12/16/2020	(Sítio Madalena)	Private rural property	F	Adult
			F	Adult
			F	Adult
			F	Adult
			F	Adult
11/3/2019	Caraguatatuba (Benfica district)	Private rural property	F	Adult
			F	Adult
			F	Adult
			M	Immature
11/2/2019	Caraguatatuba (Sítio Marisquinho)	Private rural property	F	Adult
			F	Adult
			F	Adult
	Igaratá		F	Adult
12/4/2019	(Pontal das Garças Gated	Private property		
	Community)		F	Adult
11/26/2018			F	Immature
			F	Adult
1/8/2019	Mairiporã		F	Adult
	(Cantareira State Park)	Pinheirinho trail	F	Adult
			F	Adult
1/27/2020			F	Adult
			F	Adult
			M	Immature
			M	Immature
			M	Immature
1/24/2019	Miracatu (Legado das Águas" Ecological Reserve)	Suspended trail	M	Immature
			F	Immature
			*	Immature
			*	Immature
			*	Immature
			F	Adult
			F	Adult
12/10/2019			F	Adult
			F	Adult
	Pindamonhangaba (Trabiju Municipal Park)	"Water tank" trail	F	Adult
			F	Adult
			F	Adult
12/11/2019			F	Adult
			F	Adult
			F	Adult
			F	Adult
			F	Adult
12/15/2020		Vegetation fragment south of the dirt road cutting this fragment	F	Adult
			F	Adult
	Rio Preto		F	Adult

	(Macacos Woods)		F	Adult
			F	Adult
			F	Adult
			F	Adult
			F	Adult
			F	Adult
12/18/2020	Rio Preto (Macacos Woods)	Vegetation fragment north of the dirt road cutting this fragment	F	Adult
			F	Adult
			F	Adult
			F	Adult
			F	Adult
			F	Adult
			F	Adult
			F	Adult
			F	Adult
			F	Adult
			F	Adult
			F	Adult
			F	Adult
11/26/2018	São Paulo (Cantareira State Park)	Bica trail	F	Immature
			M	Immature
1/15/2019			F	Adult
			F	Adult
			F	Adult
			F	Adult
			F	Adult
			F	Adult
9/5/2019			M	Adult
			M	Adult
9/26/2019			F	Immature
			M	Immature
9/5/2019	São Paulo (Cantareira State Park)	Administration sector	F	Immature
			M	Immature
			M	Immature
			M	Immature
			M	Immature
			M	Immature
9/26/2019			M	Immature
			F	Immature
12/18/2019			F	Adult
			F	Adult
1/15/2020			F	Adult
			M	Immature
			F	Immature
			M	Immature

*Immature forms that did not complete the developmental life cycle

Table S2. Indices of mitochondrial DNA diversity among populations (percentage of missing data tolerated < 25%)

	SP	MA	IG	RP	CA	BA	PI	MI	Mean	SD
Number of haplotypes	26	7	2	23	8	7	10	7	11.25	8.515

Nucleotide composition											
C(%)		30.97	28.4	28.57	28.71	30.35	30.79	31.14	32.48		
T(%)		29.1	29.14	31.75	30.07	30.1	29.48	28.94	25.91		
A(%)		26.05	26.42	24.6	21.65	23.38	20.96	24.29	30.66		
G(%)		13.88	16.05	15.08	19.57	16.17	18.78	15.63	10.95		
Number of loci		115	115	115	115	115	115	115	115	115	0
Number of loci used		8	14	40	35	8	31	48	3	23.375	17.121
Number of polymorphic loci		5	6	2	24	7	17	31	0	11.5	11.225
Number of gene copies	Mean	21	6.333	2	19.792	7.143	6.588	9.032	0		
	SD	0.707	0.516	0	1.503	0.378	0.507	0.706	0		
Number of alleles	Mean	2	2	2	2	2	2	2	0		
	SD	0	0	0	0	0	0	0	0		
Expected heterozygosity	Mean	0.17538	0.35079	1	0.35862	0.39456	0.40168	0.38292	0		
	SD	0.09365	0.09242	0	0.15464	0.13941	0.11801	0.12529	0		
Number of transitions		5	6	1	24	7	17	31	0	11.375	11.351
Number of transversions		0	0	1	0	0	0	0	0	0.125	0.354
Number of substitutions		5	6	2	24	7	17	31	0	11.5	11.225
Number of indels		0	0	0	0	0	0	0	0	0	0
Number of private substitutions		0	0	0	1	0	0	1	0	0.25	0.463
Theta S		1.31029	2.44898	2	6.50263	2.69972	6.93878	10.9581	0	4.10731	3.67526
SD Theta S		0.69266	1.41218	1.732	2.45405	1.47924	3.44244	4.75643	0	1.99613	1.52513
Theta		0.56615	1.66667	2	6.26087	2.21429	6.04762	9.53333	0	3.53612	3.35144
SD Theta		0.53016	1.26802	2.45	3.43962	1.55185	3.75566	5.40757	0	2.3003	1.81832

SP – São Paulo; MA – Mairiporã; IG – Igaratá, RP – Rio Preto; CA – Caraguatatuba; BA – Balsamo; PI – Pindamonhangaba; MI – Miracatu; SD – standard deviation.

Table S3. Pairwise population fixation index $\boldsymbol{F_{ST}}$ based on mitochondrial SNPs for the sampled populations of *Haemagogus leucocelaenus*.

	São Paulo	Mairiporã	Igaratá	Rio Preto	Caraguatatuba	Balsamo	Pindamonhangaba
São Paulo	-	-	-	-	-	-	-
Mairiporã	0.0201	-	-	-	-	-	-
Igaratá	0.23701	0	-	-	-	-	-
Rio Preto	0.6422*	0.57456*	0.58807*	-	-	-	-
Caraguatatuba	0.327*	0.18962*	0.12272	0.42301*	-	-	-
Balsamo	0.74982*	0.67021*	0.62904*	0.1099	0.46729*	-	-
Pindamonhangaba	0	0	0	0.5248*	0	0.54075*	-
Miracatu	0	0	1	0.5311*	0.12608	0.68457*	0

* Significance level p<0.05.

Table S4. Landscape metrics based on buffers with radii of 2,850 and 5,700 m around the collection locations.

Buffer	Municipality	Location	Land-use class														Total area (m^2)
			Water		Agricultural land use		Urban		Forest				Non-forest formation		Forestry		
			CA	PLAND	CA	PLAND	CA	PLAND	CA	PLAND	TE	ED	CA	PLAND	CA	PLAND	
2,850 m	Balsamo	i	1.8	0.0705	2033.37	79.6734	0	0	352.08	13.7955	65880	25.8137	0	0	164.88	6.4605	2552.13
	Caraguatatuba	ii	0.36	0.0141	522.45	20.46425	0	0	2030.175	79.52165	67230	26.33385	0	0	0	0	2552.985
		iii	11.34	0.4987	306.9	13.4975	859.32	37.7929	1086.3	47.7755	103140	45.361	0	0	9.9	0.4354	2273.76
	Igaratá	iv	1022.94	40.079	1153.71	45.2026	0	0	346.86	13.59	94140	36.8842	0	0	28.8	1.1284	2552.31
	Mairiporã	v	2.7	0.1058	136.53	5.3491	353.88	13.8646	2059.29	80.6805	123960	48.5661	0	0	0	0	2552.4
	Miracatu	vi	174.87	6.8507	29.52	1.1565	0	0	2348.19	91.9928	40500	15.8663	0	0	0	0	2552.58
	São Paulo	vii	4.905	0.19215	140.04	5.48575	1146.735	44.92045	1252.08	49.0473	108570	42.5297	0	0	9.045	0.35435	2552.805
		viii	4.995	0.1957	165.87	6.4978	838.035	32.8292	1534.995	60.1319	111240	43.5771	0	0	8.82	0.34555	2552.715
	Rio Preto	ix	4.365	0.171	2372.895	92.9492	36.945	1.44715	111.555	4.36975	28590	11.19905	0	0	27.135	1.0629	2552.895
		x	3.87	0.1516	2371.725	92.90665	41.625	1.63055	108.855	4.26415	28020	10.97615	0	0	26.73	1.0471	2552.805
	Pindamonhangaba	xi	0.27	0.0106	822.15	32.1995	0	0	1684.53	65.9746	161730	63.3416	0	0	46.35	1.8153	2553.3
5,700 m	Balsamo	i	16.65	0.1632	7851.15	76.9336	0	0	1276.29	12.5064	267750	26.2369	0	0	1061.01	10.3969	10205.1
	Caraguatatuba	ii	3.24	0.03175	2603.25	25.5045	0	0	7600.545	74.4638	209895	20.56375	0	0	0	0	10207.035
		iii	21.78	0.265	1255.59	15.276	1546.11	18.8106	5371.56	65.3527	257400	31.3164	0	0	24.3	0.2956	8219.34
	Igaratá	iv	2605.14	25.5267	5523.84	54.1258	53.82	0.5274	1578.6	15.4681	361650	35.4366	0	0	444.15	4.352	10205.55
	Mairiporã	v	32.94	0.3227	1217.43	11.9282	2389.77	23.4145	6562.17	64.2949	617550	60.5064	0.36	0.0035	3.69	0.0362	10206.36
	Miracatu	vi	288.36	2.8254	82.35	0.8069	0	0	9835.38	96.3678	91140	8.93	0	0	0	0	10206.09
	São Paulo	vii	9.585	0.0939	455.58	4.4636	5899.905	57.8046	3832.515	37.54925	295275	28.92975	0	0	9.045	0.08865	10206.63
		viii	13.635	0.1336	489.51	4.7961	5406.615	52.97275	4287.69	42.0098	347880	34.08445	0.135	0.00135	8.82	0.08645	10206.405
	Rio Preto	ix	21.735	0.21295	8666.595	84.9088	831.69	8.1483	595.17	5.83105	165270	16.1919	0	0	91.755	0.89895	10206.945
		x	18	0.17635	8656.56	84.812	850.95	8.33715	585.945	5.74075	163425	16.01145	0	0	91.665	0.8981	10203.12
	Pindamonhangaba	xi	20.7	0.2028	5109.57	50.0538	0	0	4859.64	47.6054	601260	58.8999	0	0	218.25	2.138	10208.16

CA –Total area; PLAND – Percentage of landscape occupied by land-use class; TE – Total forest edge (linear meters); ED –Forest edge density (m/ha); i - Sítio Madalena; ii - Sítio João; iii - Sítio Paulo; iv - Pontal das Garças Gated Community; v - Pinheirinho trail; vi - Legado das Águas Ecological Reserve; vii - Bica Trail; viii - Administrative Sector; ix - Macacos forest (fragment south of dirt road); x - Macacos Forest (fragment north of dirt road); and xi - Trabiju Municipal Park.

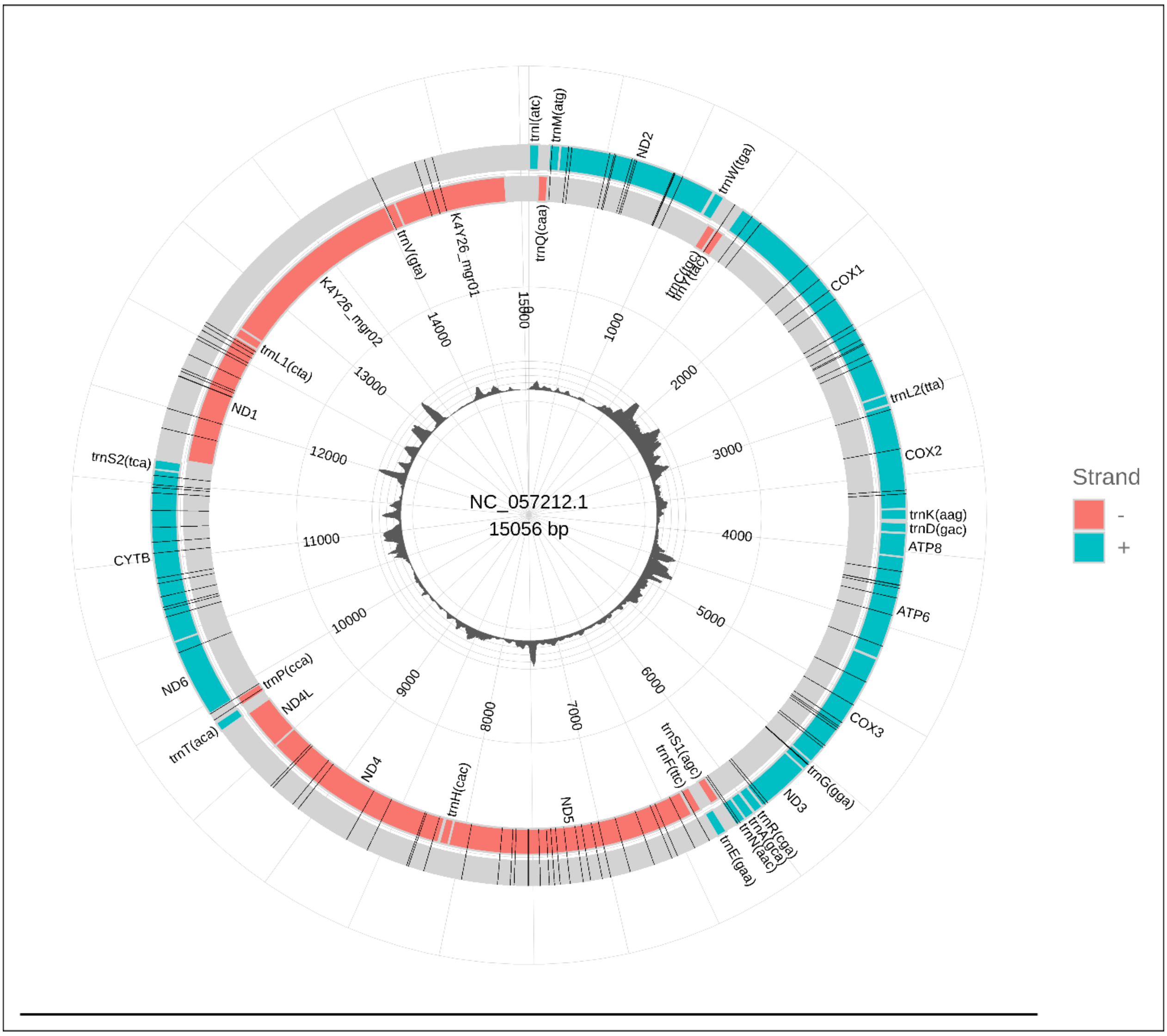

Figure S1. Map of the *Hg. leucocelaenus* reference mitochondrial genome (NC_057212.1). Variant calling identified a total of 115 SNPs (represented by the black bars). These are distributed along the mitochondrial genome, and the area where they were identified is represented by the center of the graph.

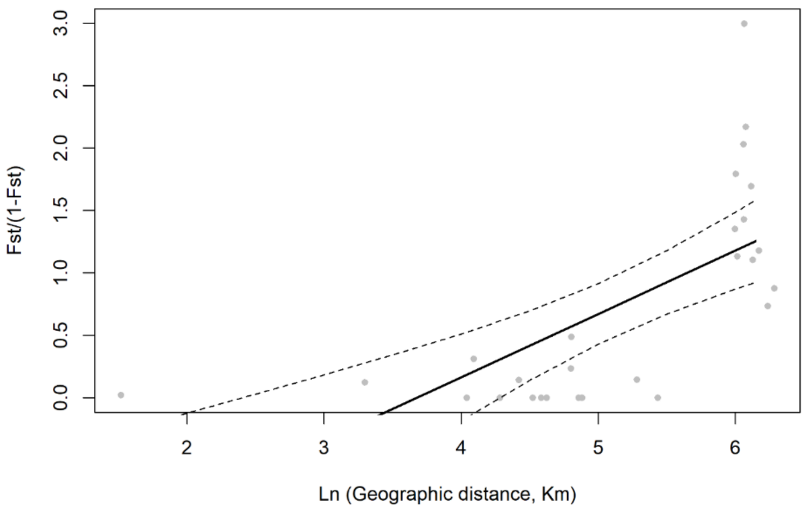

Figure S2. Linear correlation on a natural log scale between genetic distance (F_{ST} $/(1 - F_{ST}$) and geographic distance separating the *Hg. leucocelaenus* populations studied. Gray points represent the distribution of the data, and the black line represents the model fit, while the dashed lines represent the 95% confidence interval.

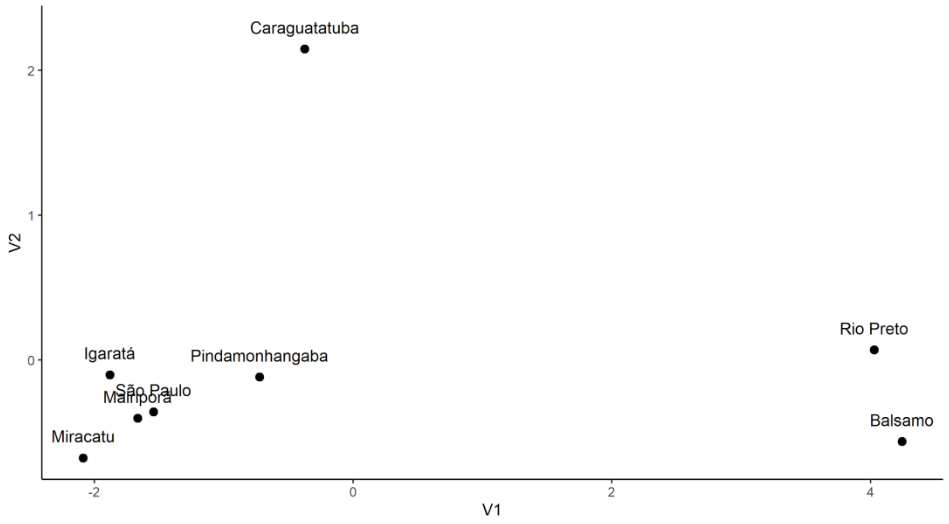

Figure S3. Multidimensional scaling analysis (MDS) of the Euclidian distances between the allele frequencies of the populations for the mitochondrial SNPs.

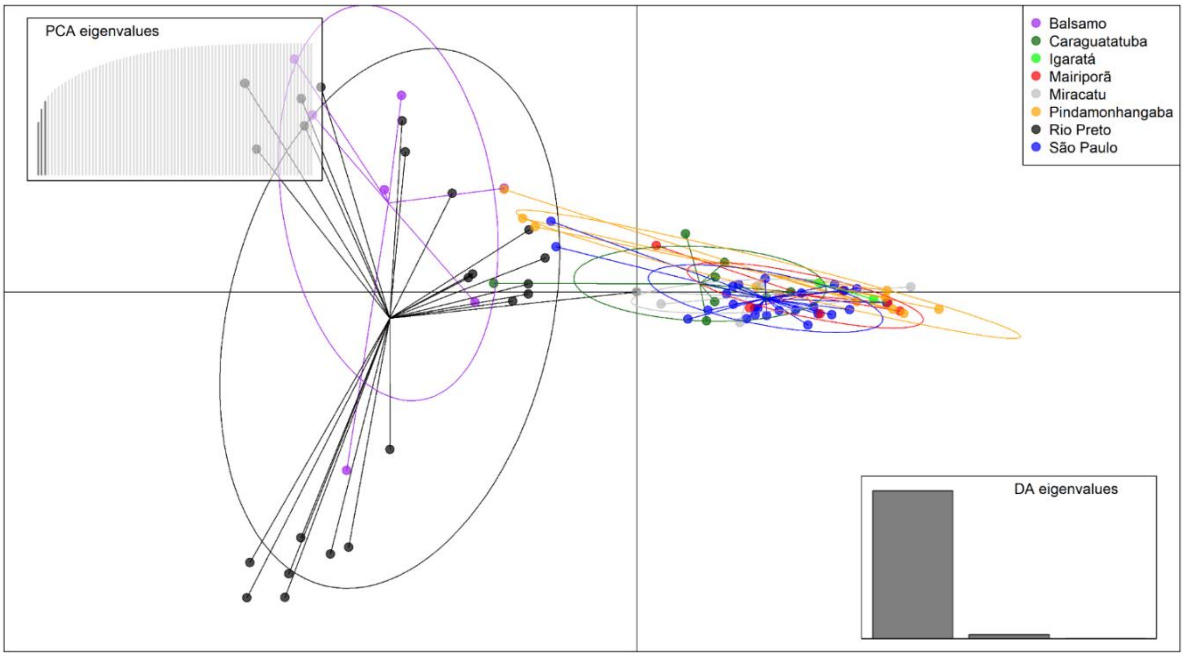

Figure S4. Discriminant analysis of principal components (DAPC) of individuals sampled in eight municipalities.

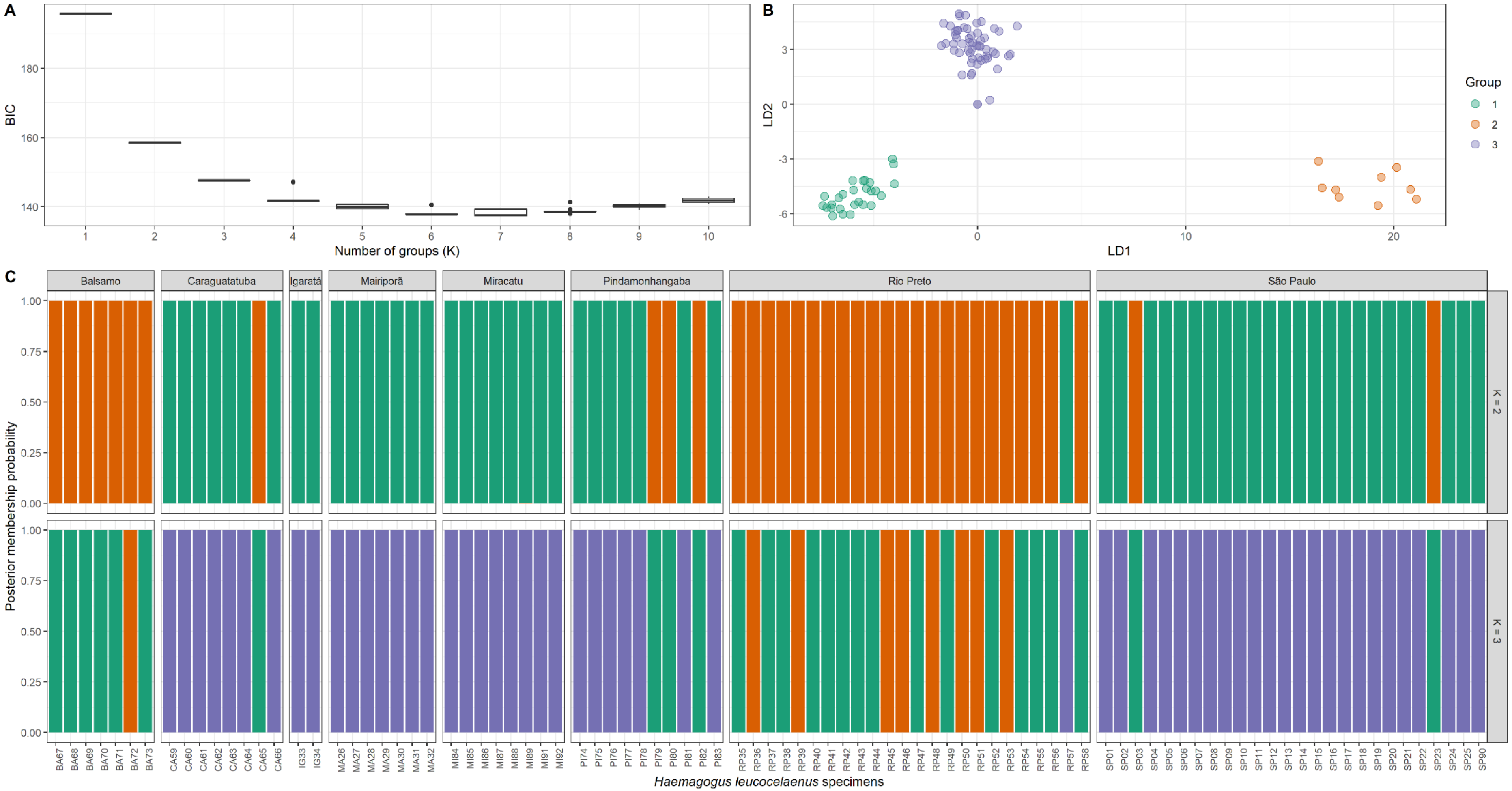

Figure S5. Multipanel figure showing population structure of sampled *Hg. leucocelaenus* specimens. A and B: number of genetic clusters (K); C: subdivision of specimens for K=2 and K=3. Each of the 92 specimens of the 8 populations sampled is represented by a vertical line.

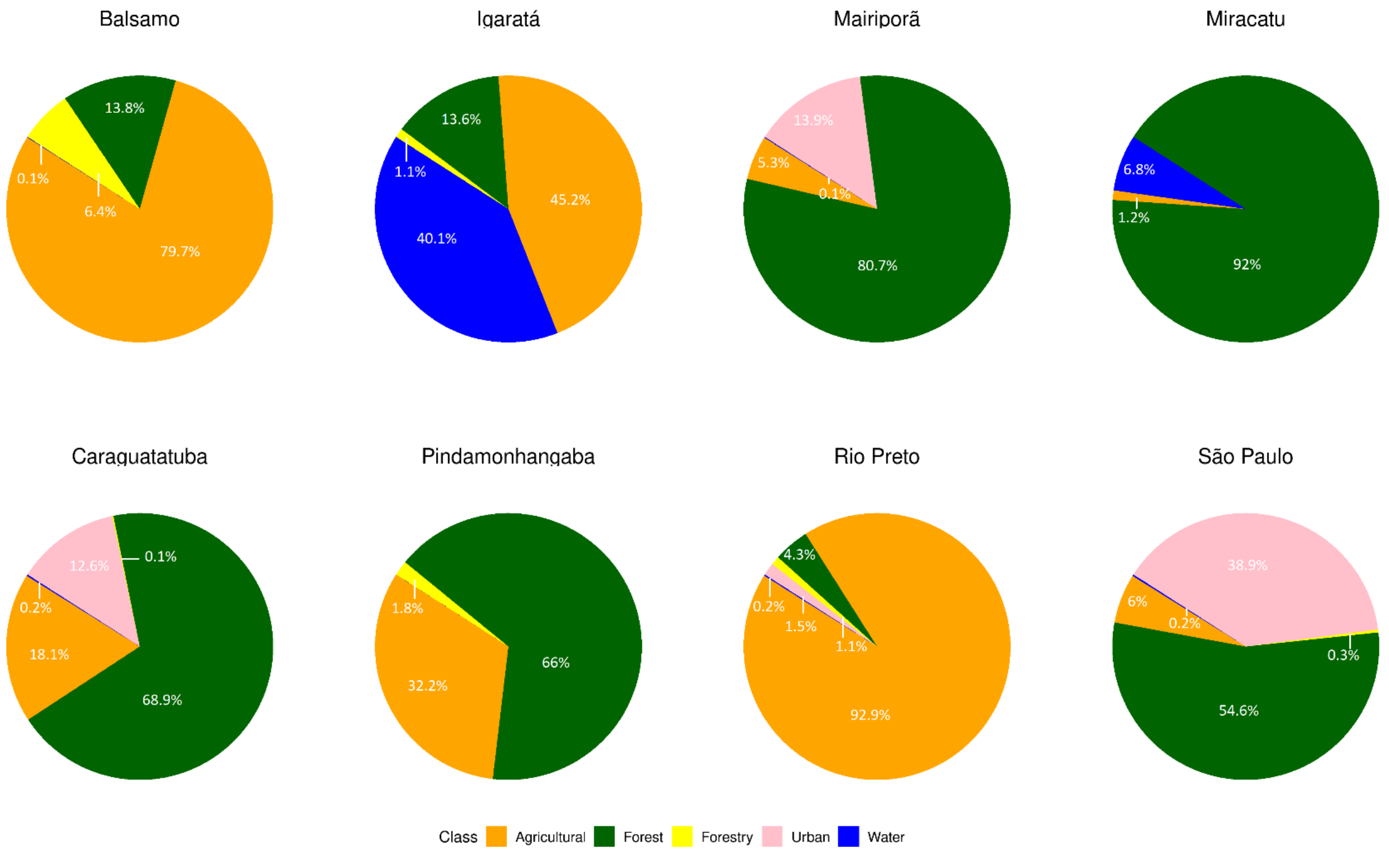

Figure S6. Landscape configuration based on 2,850 m buffers around the collection points - half the maximum species dispersal distance for *Hg. leucocelaenus* [64]. This is in accordance with the dispersal radius expected by Jackson & Fahrig [63] for a given species.

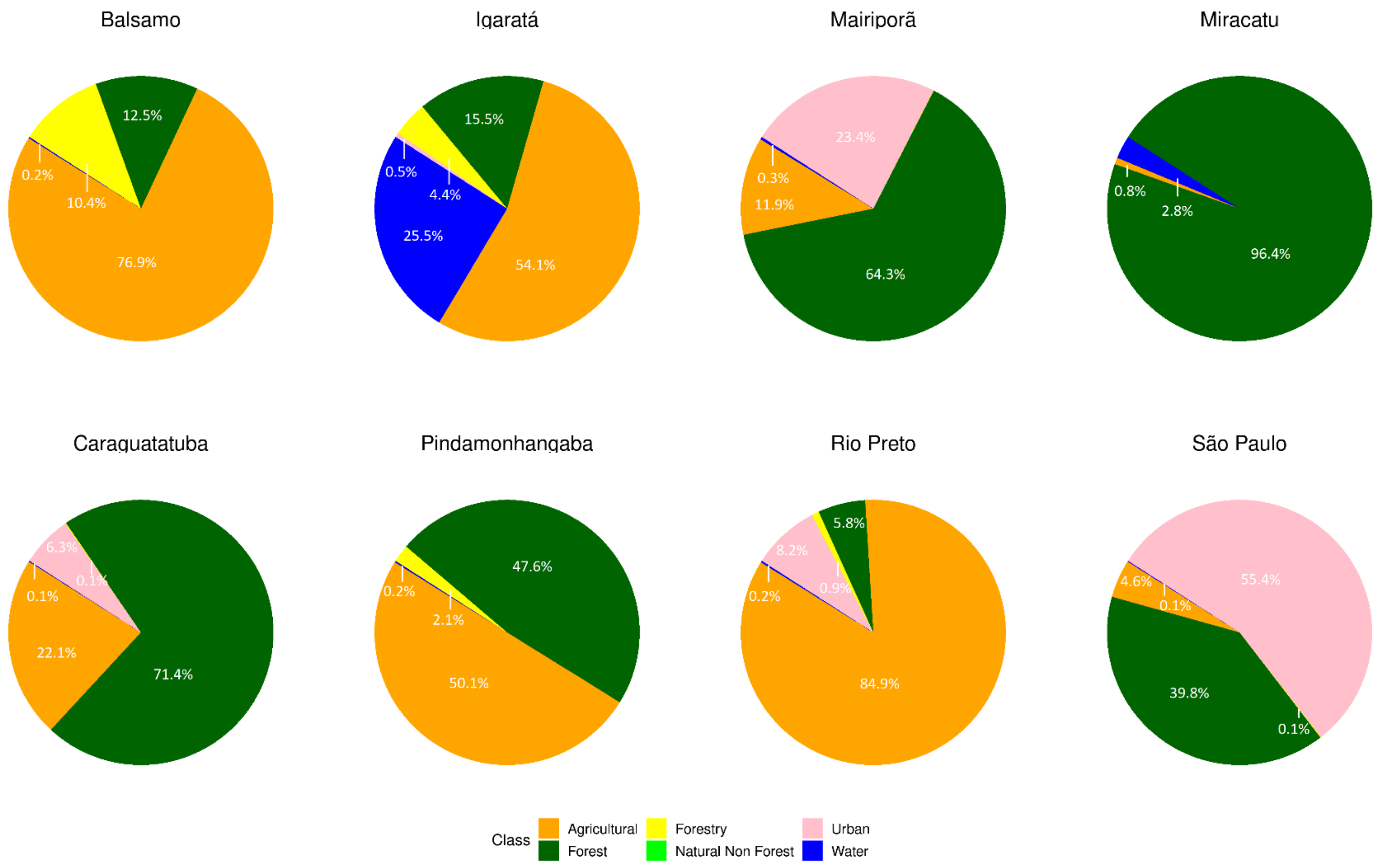

Figure S7. Landscape configuration based on 5,700 m buffers - the maximum species dispersal distance for *Hg. leucocelaenus* [64].
